# Mendelian randomization study revealed a gut microbiota-immune system-kidney junction axis in chronic kidney disease

**DOI:** 10.1038/s41598-025-05941-x

**Published:** 2025-07-01

**Authors:** Junjie Tan, Zhile Xiong, Shengyou Yu, Wei Lu, Li Yu

**Affiliations:** 1 Department of Pediatrics, The First Affiliated Hospital, Jinan University, Guangzhou, 510630 Guangdong Province China; 2 Department of Pediatrics, Qingyuan Maternal and Child Health Hospital, Qingyuan, 511510 Guangdong Province China; 3 Institute of Microbiology, Infectious Diseases and Immunology, Charité – Universitätsmedizin Berlin, corporate member of Freie Universität Berlin and Humboldt- Universität zu Berlin, Hindenburgdamm 30, 12203 Berlin, Germany

**Keywords:** Microbiome, CKD, eGFR, UACR, Mendelian randomization, Immune cells, Mediator analysis, Clinical microbiology, Microbial communities, Immunology, Microbiology, Nephrology

## Abstract

The alterations of the gut microbiome and cytokine profiles and an elevated risk has correlated with kidney disease progression. However, the causal relationship between gut microbiota and chronic kidney disease (CKD) or related kidney function, and whether cytokines and immune cells act as mediators, remains unclear. Using genome-wide association studies (GWAS) data for CKD, estimated glomerular filtration rate (eGFR) and UACR (urinary albumin to creatinine) from the CKDGen consortium, microbiome data from the MiBioGen consortium and the Dutch Microbiome Project (DMP), 41 cytokine and 731 immune cell traits were identified from large-scale GWAS summary data. We performed two-sample Mendelian randomization (MR) analysis to analyses the causal relationships between gut microbiome, circulating cytokines, immune cells and CKD, eGFR and UACR. In addition, we investigated whether cytokines and immune cells are the mediating factor in the pathway from gut microbiome to CKD, eGFR and UACR. We demonstrated the causal relationships between 8 gut microbiotas in MiBioGen and 8 gut microbiota and 6 metabolism pathways in DMP with CKD, 7 gut microbiotas in MiBioGen and 7 gut microbiota and 3 metabolism pathways with eGFR and 4 gut microbiotas in MiBioGen and 10 gut microbiota and 3 metabolism pathways in DMP with UACR. Additionally, we identified 25 cytokine and immune cell characteristics associated with CKD, 18 with eGFR and 22 with UACR. Importantly, we identified no cytokine, but several immune cell properties that mediate the effects of microbiome on CKD, eGFR and UACR through mediation MR analysis. For instance, *Alistipes indistinctus* and *Alistipes putredinis* affects CKD via CD28 + CD45RA + CD8 + T cell. The mediation effects highlighted the intricate relationship between gut microbiome exposure, immune cell activity, and their combined influence on CKD. This data supports a causal effect of the gut microbiome on CKD, eGFR and UACR and underscores the value of MR in clarifying causal relationships identified in microbiome-wide association studies. Circulating immune cells may act as mediators in the pathway linking gut microbiota to CKD progression.

**Supplementary Information:**

The online version contains supplementary material available at 10.1038/s41598-025-05941-x.

## Introduction

Chronic kidney disease (CKD) is a progressive condition marked by structural and functional changes in the kidneys^1^. It is defined by a sustained estimated glomerular filtration rate (eGFR) of less than 60 ml per minute per 1.73 m² of body surface area, or urinary albumin excretion of 30 mg or more per day, or both, for a duration of at least three months^2^. An elevated urinary albumin to creatinine ratio (UACR) is also used to diagnose and stage CKD^3^. Numerous studies indicate that CKD is an increasingly important global health problem, with its prevalence and incidence rising and now affecting more than 800 million people worldwide^4,5^. A systematic analysis from the Global Burden of Disease Study revealed a 41.5% increase in global mortality from CKD between 1990 and 2017, leading to approximately 1.23 million deaths in 2017^6^. In addition, results from a Global Burden of Disease study in China show a notable increase in CKD prevalence from 6.7 to 10.6% and mortality from 8.3/100,000 to 13.8/100,000 between 1990 and 2019^7^. Besides, it is also projected that CKD disease will be the 5th leading cause of death globally, representing one of the largest expected increases among major causes of death by the end of 2040^8^. As the progression of CKD is irreversible, understanding its etiology is crucial for prevention and for identifying new treatment options based on the underlying causes of the disease.

Regardless of the underlying cause, CKD is often associated with chronic inflammation that both drives and results from glomerular and tubulointerstitial damage^9^. The potential for inflammation to serve as a target for intervention in CKD is an area of growing interest, particularly with regard to the various cytokines and immune cells that play a role in this process. Prior research has demonstrated that inflammation is a key factor in the deterioration of kidney function across nine distinct forms of CKD in European populations^10^. Patients with different forms of CKD, even those with asymptomatic proteinuria, experience a unique microinflammatory state compared to individuals with normal kidney function^11^. Inflammation serves as a critical link between pathological changes in the glomeruli and tubules.

The human gut microbiome, comprising over 500 microbial species, plays a key role in energy homeostasis and maintaining gut barrier integrity^12,13^. Changes in microbial composition have been linked to the pathogenesis and progression of various conditions, including metabolic disorders, autoimmune diseases, and cancers^14,15^. Emerging evidence highlights the involvement of the gut microbiota in renal injury and fibrosis. For instance, the gut microbiome has been shown to regulate the production of hyperglycosylated IgA1 via the TLR4 signaling pathway^16,17^. In addition, *Lactobacillus*. spp such as *Lactobacillus johnsonii* have been demonstrated protective effects in membranous nephropathy, and alterations in the gut microbiome have been observed in kidney transplant recipients^18,19^. Moreover, recent studies suggest that patients with CKD may exhibit a distinct intestinal microbiome and microbiome imbalance^20^. Alterations in the gut microbiota, their functions, and the production of metabolites that lead to uremic toxicity are believed to be linked to the onset and progression of CKD. Damage to the intestinal mucosa allows specific microbes to access lymph nodes and the spleen, where they activate particular immune cells and cytokines, triggering a systemic immune response.

Since microbiome characteristics, circulating cytokines, and immune cells offer potential for CKD intervention, understanding the relationship between the microbiome, cytokines, immune cells, and CKD, as well as its effects on renal function, is crucial.

Mendelian randomization (MR) uses genetic variants as proxies to infer causal relationships, helping to bypass confounding biases and evaluate the causal effect between exposures and outcomes. The two-sample MR approach, which leverages published summary statistics from large-scale genome-wide association studies (GWAS), provides greater statistical power for identifying these causal links. Recent MR analyses have provided fresh insights into the therapeutic potential of targeting the microbiome and cytokines in CKD by establishing a causal association between certain microbial taxa and CKD risk. These findings not only underscore the role of gut microbiota in CKD progression but also highlight the potential for microbiome-targeted therapies. By identifying specific microbial strains and their interactions with immune pathways, this research opens new avenues for personalized treatments that could modify the course of CKD and reduce its burden^21,22^. But the underlying mechanism for immune system is still unknown. The aim of this study was to conduct a comprehensive MR analysis to explore the causal effects between the gut microbiome, 41 cytokines, 731 immune cells. Then we explored whether cytokines and 731 immune cells as mediators in the pathway from gut microbiota to chronic kidney disease and renal function.

## Method

### Study design

**Fig. 1:**
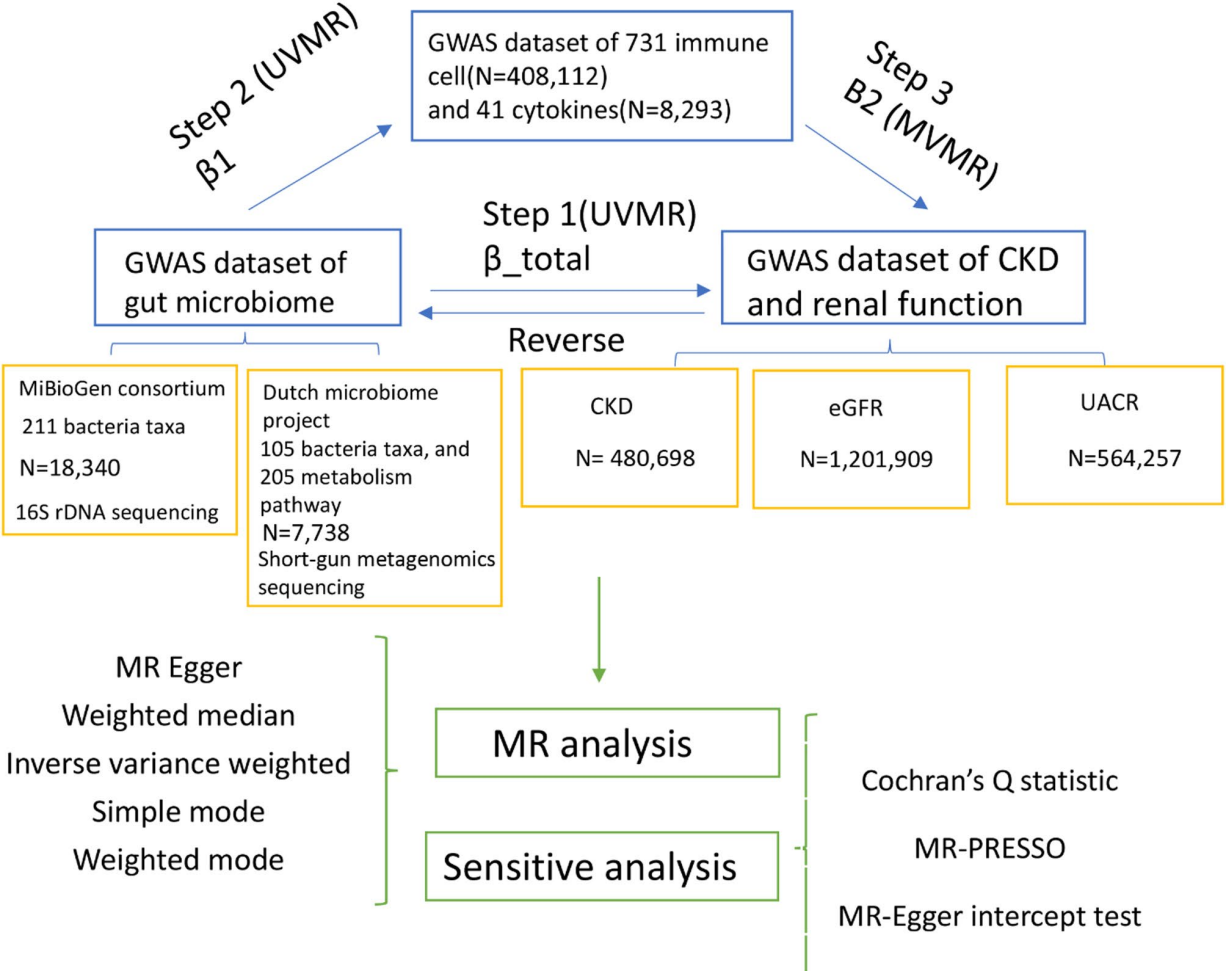
The study design. A two-step Mendelian randomization study of gut microbiota on CKD, eGFR, and UACR mediated by immune cell and cytokine. CKD: chronic kidney disease; eGFR: estimated glomerular filtration rate; UACR: urinary albumin to creatinine. MR, Mendelian randomization; MVMR, multivariable Mendelian randomization; UVMR, univariable Mendelian randomization.

The outline of bidirectional two-sample MR analysis with gut microbiome on CKD, eGFR, UACR and mediator analysis (41 cytokines and 731 immune cells) was shown in Fig. 1. Step 1: Bidirectional two-sample MR analysis: analysis of causal effects 403 bacterial taxa and 205 metabolism pathways on CKD, eGFR and UACR; Step 2: Mediator analysis: Analysis of causal effects of 731 immune cells and 41 cytokines on CKD, eGFR and UACR; Step 3: Mediation analysis of cytokines and immune cells from gut microbiome to CKD, eGFR and UACR. We defined single-nucleotide polymorphisms (SNPs) as IVs.

MR analysis is based on three core assumptions. Firstly, that the IVs are closely associated with the exposure factors; Secondly, that the IVs are not associated with confounding factors; and thirdly, that the IVs do not directly affect the outcome, but rather exert an influence upon it indirectly through the exposure. All data sources of the MR database used in this study are listed in Table S1.

### Data source

#### Exposure GWAS data for Microbiome

The exposure GWAS data for the gut microbiome was obtained from the two GWAS database: MiBioGen consortium and Dutch microbiome project (DMP) database^23,24^. (1) The MiBioGen consortium database encompasses a total of 211 gut microbiota taxa, encompassing 9 phyla, 16 classes, 20 orders, 35 families, and 131 genera, derived from 16 S rDNA sequencing from 18,340 individuals across 24 cohorts. We removed 15 unknown families of genera and included 196 taxa for MR analysis (2). Data are available at https://mibiogen.gcc.rug.nl/. The Dutch Microbiome Project (DMP) encompasses 5 phyla, 10 classes, 13 orders, 26 families, 48 genera, and 105 species of gut microbiota abundance, as well as 205 gut bacterial pathways abundance. These data were obtained through shotgun metagenomic sequencing of 7,738 European-ancestry individuals. The relevant accession numbers are GCST90027446 to GCST90027857. Totally, the comprehensive MR analysis encompassed a total of 403 bacterial taxa and 205 metabolic pathways derived from the MiBioGen consortium and DMP (Table S2).

#### Outcome GWAS data for CKD, eGFR and UACR

The GWAS summary data of CKD, eGFR and UACR were obtained from the Chronic Kidney Diseases Genetics Consortium (CKDGen consortium) and are available for download at http://ckdgen.imbi.uni-freiburg.de/ (accessed on September 15, 2024). The GWAS summary statistics of IVs for CKD were derived from the CKDGen Consortium, which included 23 European ancestry cohorts (439,303 controls and 41,395 patients; *n* = 480,698)^25^. Furthermore, the GWAS summary statistics of eGFR were derived from a meta-analysis that incorporated data from the CKDGen (*n* = 1,201,909)^26^. The GWAS data pertaining to UACR were obtained from a meta-analysis that recorded the summary data of trans-ethnic (*n* = 564,257) and European-ancestry populations^27^.

#### GWAS data for mediator analysis in cytokine and immune cells

In this study, mediation analysis was employed by using GWAS summary data pertaining to 731 immune cell phenotypes and 41 inflammatory cytokines. With regard to circulating cytokines, GWAS data for 41 inflammatory factors were obtained from meta-analyses involving 8,293 individuals from 3 independent population cohorts. The Young Adults Study (*N* = 1980) and the FINRISK survey (FINRISK1997(*N* = 4608) and FINRISK2002(*N* = 1705))^28^. The relevant accession numbers are GCST004420-GCST004460. For the circulating immune cell database, we obtained the 731 immune cell phenotype data included 3,757 individuals of European ancestry from non-overlapping cohorts: absolute cell counts (AC, *n* = 118), median fluorescence intensity reflecting surface antigen levels (MFI and SAL, *n* = 389), morphological parameters (MP, *n* = 32), and relative cell counts (RC, *n* = 192)^29^. The MFI, AC, and RC features encompass various immune cell types, including B cells, CDCs, mature stages of T cells, monocytes, myeloid cells, TBNK (T cells, B cells, natural killer cells), and Treg panels. The MP feature incorporates both CDC and TBNK panels. The aforementioned immunophenotypes furnish invaluable insight into the correlation between immune cell attributes and osteoporosis. The relevant accession numbers are GCST0001391-GCST0002121. All of the datasets were derived exclusively from European populations.

#### Instrumental variables selection

Firstly, we selected the single-nucleotide polymorphisms (SNPs) with significant associations for gut microbiota taxa and gut metabolism pathway in MiBioGen consortium and DMP database (*P* < 1 × 10– ^5^). Subsequently, the SNPs with significant associations for 731 immune cells and 41 cytokines were also selected. In order to maximize the number of available instruments for each cytokine and immune cells phenotype, the SNPs with a P-value of less than 5 × 10^− 6^ were selected in the cytokine dataset and a P-value of less than 1 × 10– ^5^ in the immune cell dataset. Secondly, the selected instrumental variables in gut microbiota taxa and gut metabolism pathway, 731 immune cells and 41 cytokines need to meet the independence test. We excluded the SNPs with linkage disequilibrium (LD) in the analysis The LD of the selected SNPs with a strong correlation to the gut microbiota should satisfy the criteria of a correlation coefficient (r²) of less than 0.001 and a distance greater than 10,000 kilobases (kb). Thirdly, a crucial aspect of MR analysis is to ascertain that the effects of (SNPs) on exposure are attributable to the same allele as the effects on the outcome. Following the matching of the outcome, palindromic SNPs were removed. The relevant information was extracted, comprising chromosome, effect allele (EA), other allele (OA), effect allele frequency (EAF), effect sizes (β), standard error (SE), and p-value. Lastly, the explained variance (R²) and F-statistic parameters were calculated to ascertain whether the identified IVs were significantly associated with exposure. Furthermore, SNPs with F-values below 10 (equations: To ensure the strength of the association between the instrumental variables and the exposure factors, the following equation is used: F = [R²/K × (N-K-1)/(1-R²)] or F = b² - exposure/SE² - exposure^30^.

### MR analysis

#### Primary analysis

To estimate the causal effects of each gut microbiota, gut metabolism pathway, cytokines and immune cells on CKD, eGFR and UACR, we performed two-sample MR analysis (Fig. 1). Inverse variance weighted (IVW), weighted median (WM), MR-Egger approaches, Simple mode, Weighted mode, and Wald ratio were used to investigate the causal effect of gut microbiota, gut metabolism pathway, 41 cytokines and 731 immune cells on CKD, eGFR and UACR. IVW were used as the primary approach to the MR results to select the positive casual effect^31^. The MR results were included with the corresponding odds ratios (ORs) and 95% confidence intervals (CI). The results were deemed statistically significant when the P-value of the IVW was less than 0.05 and the direction of the IVW and MR-Egger were consistent. A two-sided P-value that passed the Bonferroni correction was defined as statistically significant, with a threshold of 0.0012 (0.05/41) for cytokines. A P-value of less than 0.05, but above the Bonferroni-corrected threshold, was considered to provide suggestive evidence of an association.

#### Mediation analysis

The two-sample MR analysis (Fig. 1) identified the gut microbiota taxa, gut microbiota metabolism pathway, cytokines and immune cells with significant causal effects on CKD, eGFR and UACR for inclusion in the mediation analysis. The initial objective was to ascertain whether there was a causal effect of the gut microbiota on cytokines and immune cells (UVMR). Should this be confirmed, a subsequent step would be to perform multiple MR analysis (MVMR) to determine whether cytokines and immune cells constituted the mediation factors in the pathway from gut microbiota to CKD, eGFR and UACR. To evaluate the potential bidirectional causal relationships between the gut microbiota, cytokines, immune cells and CKD, eGFR and UACR, we employed a two-sample MR analysis (Fig. 1), wherein CKD, eGFR and UACR were designated as the “exposure” variables and the gut microbiota, cytokines and immune cells associated with CKD, eGFR and UACR were designated as the “outcome” variables. The single-nucleotide polymorphisms (SNPs) that were significantly associated with CKD, eGFR and UACR (*P* < 5 × 10⁻⁸) were selected as IVs.

#### Sensitivity analysis

Cochran’s Q test was employed to assess the heterogeneity of each SNP, representing the associations between the SNPs and both the exposures and outcomes. Furthermore, the potential for horizontal pleiotropy was evaluated using the MR-PRESSO and MR-Egger regression methods. The MR-PRESSO method was employed to identify any significant outliers and to correct for the potential horizontal pleiotropy effect by removing these outliers. All analyses were conducted using the R studio software (version 4.2.2). The MR analysis was conducted using the R-based “TwoSampleMR” package. The MR-PRESSO package was employed for multiplicity testing.

#### Ethics

The present study constitutes a secondary analysis of publicly available GWAS summary statistics. Ethical approval was obtained for each of the original GWAS studies. Furthermore, no individual-level data were employed in the course of this study. Accordingly, no further ethical review board approval was necessary.

## Results

### IVs selection

Firstly, from the MiBioGen consortium dataset, we identified, 142, 223, 279, 380, and 1531 SNPs associated with 196 gut microbiota taxa at the phylum, class, order, family, genus levels, respectively (*P* < 1 × 10– ^5^) (Table S2 order1). Secondly, we identified 1923 SNPs and 2117 SNP selected as IVs for the 105 gut microbiota taxa and gut microbiome metabolism pathway at a level of *P* < 1 × 10– ^5^ from DMP (Table S2 order 2). Subsequently, 451 SNPs were identified as being associated with 41 cytokines at a level of *P* < 5 × 10– ^6^ (Table S2, order 3), while 18,971 SNPs were identified as being associated with 731 immune cells at a level of *P* < 1 × 10– ^5^ (Table S2, order 4).

### Causal effects of gut microbiota on CKD and its related renal function

#### MR analysis of gut microbiota on CKD

**Fig. 2:**
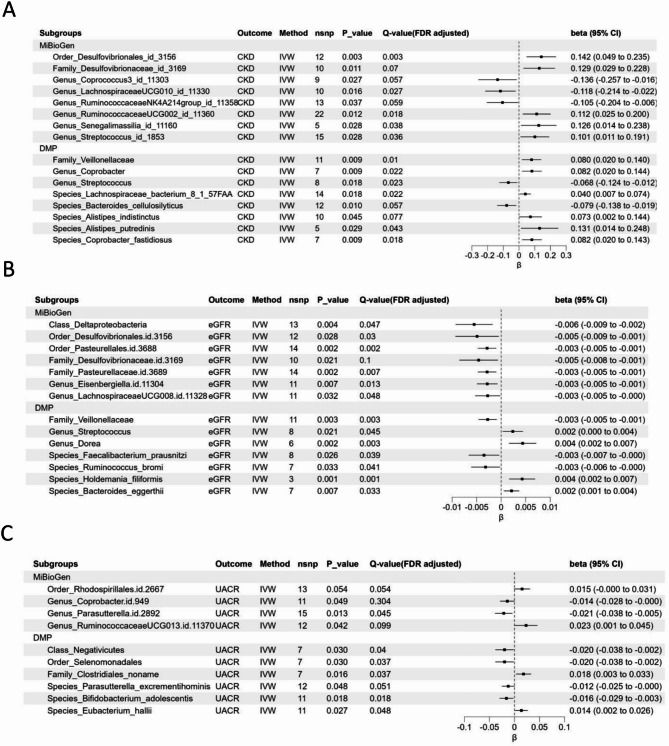
**(A)** Results of Bi-directional Univariate Mendelian Randomization the gut microbiota and CKD. **(B)** Results of Bi-directional Univariate Mendelian Randomization the gut microbiota and eGFR. **(C)** Results of Bi-directional Univariate Mendelian Randomization the gut microbiota and UACR. CKD: chronic kidney disease; eGFR: estimated glomerular filtration rate; UACR: urinary albumin to creatinine; IVW, inverse variance weighted; CI, confidence interval.

In MiBioGen database, a total of 8 gut microbiota (including 1 order, 1 family, and 6 genera) were associated with CKD after initial MR analysis and take as suggested casual bacterial taxa. In the DMP database, a total of 8 gut microbiotas (including 1 order, 2 family, 3 genera, and 6 species) were associated with CKD after MR analysis with IVW as the primary method and take as suggested casual bacterial taxa (Fig. 2A and Table S3(order 1)).

In Fig. 2A, after IVW analysis and Bonferroni correction and when FDR-corrected P value were < 0.05, we found that in the MiBioGen database, order *Desulfovibrionales* (β: 0.14201, 95% CI: 0.04940 to 0.23462, *P* = 0.003, FDR-corrected *P* = 0.003, genus *Ruminococcaceae*UCG002 (β: 0.11231, 95% CI: 0.02461 to 0.2001,*P* = 0.012, FDR-corrected P value:0.018), genus *Senegalimassilia* (β: 0.12563, 95% CI: 0.01375 to 0.23752, *P* = 0.028, FDR-corrected P value:0.038), and genus *Streptococcus* (β: 0.10079, 95% CI: 0.01080 to 0.19077, *P* = 0.028, FDR-corrected P value:0.036) are positive correlated with CKD, while genus *Lachnospiraceae*UCG010 (β: -0.11833, 95% CI: -0.21424 to -0.02242,*P* = 0.016, FDR-corrected P value:0. 027) were negative correlated with CKD. In DMP, family *Veillonellaceae* (β: 0.07993, 95% CI: 0.01980 to 0.14007, *P* = 0.009, FDR-corrected *P* = 0.01), genus *Coprobacter* (β: 0.08201, 95% CI: 0.0205 to 0.14354, *P* = 0.012, FDR-corrected *P* = 0.018), species *Lachnospiraceae* bacteria (β: 0.04043, 95% CI: 0.0069 to 0.07394, *P* = 0.018, FDR-corrected P value:0.022), species *Alistipes putredinis* (β: 0.13095, 95% CI: 0.01367 to 0.24824, *P* = 0.029, FDR-corrected P value:0. 043), and species *Coprobacter fastidiosus* (β: 0.08176, 95% CI: 0.02031 to 0.14321, *P* = 0.009, FDR-corrected P value:0.018) were positive correlated with CKD, while genus *Streptococcus* (β: -0.06775, 95% CI: -0.12368 to -0.01183, *P* = 0.018, FDR-corrected *P* = 0.023) were negative correlated with CKD.

Moreover, family *Desulfovibrionaceae* id_3169, genus *Coprococcus*3_id_11303 in MiBioGen and species *Bacteroides cellulosilyticus* and species *Alistipes indistinctus* in DMP have casual effect with CKD after first MR analysis but q-value were higher than 0.05 after Bonferroni correction (Fig. 2A and Table S3(order 1)).

#### MR analysis of gut microbiota on eGFR

In MiBioGen database, a total of 7 gut microbiotas (including, 1 class, 2 order, 2 family, and 2 genera) were associated with eGFR (Additional file 3: Table S3(order 1), Fig. 2). In Dutch microbiome project database bacteria part, a total of 7 gut microbiotas (including 1 family, 3 genera, and 6 species) were associated with eGFR.

In Fig. 2B, after IVW analysis and Bonferroni correction and when FDR adjusted p-val were < 0.05, we found that in the MiBioGen database Class *Deltaproteobacteria*, Order *Desulfovibrionales*.id.3156, Order *Pasteurellales*.id.3688, Family *Pasteurellaceae*, Genus *Eisenbergiella*.id.11,304, Genus *Lachnospiraceae*UCG008 were positive correlated with eGFR. In DMP, Genus *Streptococcus*, Genus *Dorea*, Species *Faecalibacterium prausnitzi*, Species *Ruminococcus bromi*, Species *Holdemania filiformis*, Species *Bacteroides eggerthii* were positive correlated with eGFR While Family *Veillonellaceae Ruminococcus bromi*, *Faecalibacterium prausnitzi* were negative correlated with eGFR.

#### MR analysis of gut microbiota on UACR

In consortium database, a total of 4 gut microbiotas (including 1 order, and 3 genera) were associated with UACR (Additional file 3: Table S3(order 1), Fig. 2). In Dutch microbiome project database bacteria part, a total of 10 gut microbiotas (including 1 class, 1 order, 1 family, 2 genera, and 5 species) were associated with UACR.

In Fig. 2C, after IVW analysis with FDR-corrected *P* < 0.05, we found that in the MiBioGen database Order_Rhodospirillales.id.2667, Genus_Parasutterella.id.2892, have a significant causal effect with UACR. In DMP, Class *Negativicutes*, Order *Selenomonadales*, Family *Clostridiales_noname*, Species *Parasutterella excrementihominis*, Species *Bifidobacterium adolescentis*, Species *Eubacterium hallii*.

#### MR analysis of gut microbiota metabolism pathways on CKD, eGFR and UACR

**Fig. 3:**
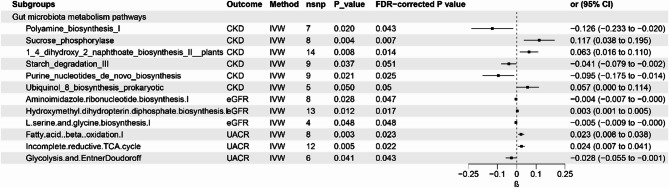
Results of Bi-directional Univariate Mendelian Randomization the gut microbiota metabolism and CKD, eGFR and UACR. CKD: chronic kidney disease; eGFR: estimated glomerular filtration rate; UACR: urinary albumin to creatinine; IVW, inverse variance weighted; CI, confidence interval.

In DMP database gut microbiota metabolism pathway’s part, a total of 6 metabolism pathways has causal relationship with CKD and 3 metabolism pathways with eGFR and 3 metabolism pathways were associated with UACR. The detailed of MR analysis were given at Fig. 3.

#### Reverse MR analysis of gut microbiota and gut microbiota metabolism pathways on CKD, eGFR and UACR

Finally, reverse MR analysis was performed for the bacterial taxa presenting significant results with CKD, eGFR and UACR. After additional analysis and sensitivity analysis, no significant and stable results were found (Table S7 and S8 (order 1 to 3)).

### Causal effects of cytokines and 731 immune cells features on CKD, eGFR and UACR

#### MR analysis of cytokine and 731 immune cells on CKD

**Fig. 4:**
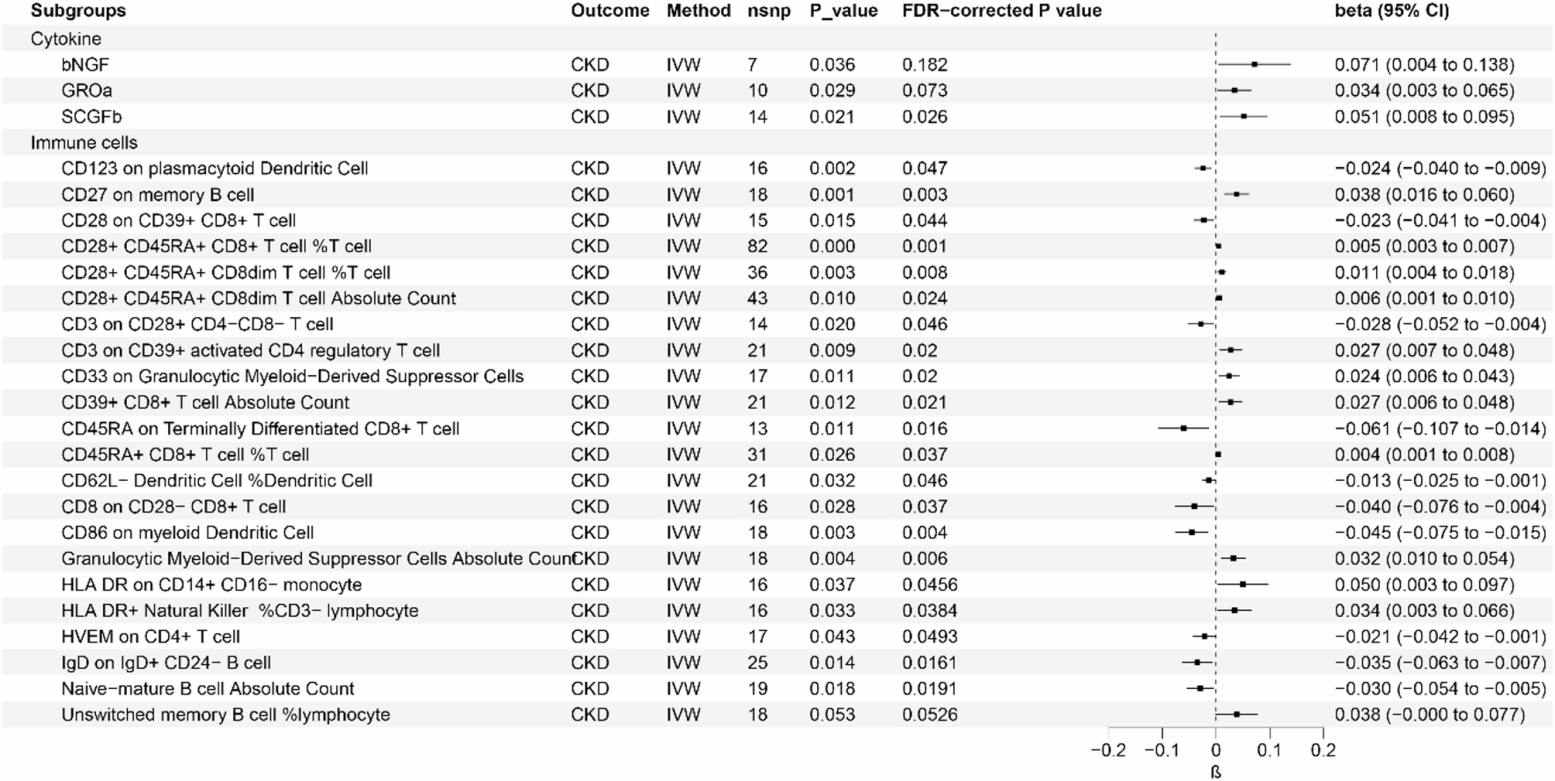
Results of Bi-directional Univariate Mendelian Randomization on the interplay between the mediators (immune cells and inflammatory cytokines) and CKD. CKD: chronic kidney disease; eGFR: estimated glomerular filtration rate; UACR: urinary albumin to creatinine; IVW, inverse variance weighted; CI, confidence interval.

As shown in Fig. 4, cytokines including bNGF, GROa and SCGFb and 22 immune cells feature had causal relationship with CKD. In Fig. 4a, we found that CD27 on memory B cell, CD28 + CD45RA + CD8 + T cell %, CD28 + CD45RA + CD8dim T cell %, CD28 + CD45RA + CD8dim T cell Absolute Count, CD3 on CD39 + activated CD4 regulatory T cell, CD33 on Granulocytic Myeloid-Derived Suppressor Cells, CD39 + CD8 + T cell Absolute Count, CD45RA + CD8 + T cell %, Granulocytic Myeloid-Derived Suppressor Cells Absolute Count, HLA DR on CD14 + CD16- monocyte, HLA DR + Natural Killer %CD3- lymphocyte and Unswitched memory B cell %lymphocyte were positive correlated with CKD, while CD123 on plasmacytoid Dendritic Cell, CD28 on CD39 + CD8 + T cell, CD3 on CD28 + CD4-CD8- T cell, CD45RA on Terminally Differentiated CD8 + T cell, CD62L- Dendritic Cell %Dendritic Cell, CD8 on CD28- CD8 + T cell, CD86 on myeloid Dendritic Cell, HVEM on CD4 + T cell, IgD on IgD + CD24- B cell, and Naive-mature B cell Absolute Count were negative correlated with CKD.

#### MR analysis of cytokine and 731 immune cells on eGFR

As shown in supplemental Fig. 1, 1 cytokine IL4 and 18 immune cells feature have causal relationship with eGFR. CD123 on CD62L + plasmacytoid Dendritic Cell, CD123 on plasmacytoid Dendritic Cell, CD24 on IgD- CD38- B cell, CD24 on transitional B cell, CD3 on Effector Memory CD4 + T cell, CD45RA- CD4 + T cell %, CD62L- plasmacytoid Dendritic Cell %, HVEM on Central Memory CD8 + T cell and Switched memory B cell Absolute Count were positive correlated with eGFR, while BAFF-R on IgD + CD38- unswitched memory B cell, BAFF-R on switched memory B cell, CD27 on IgD- CD38dim B cell, CD27 on switched memory B cell, CD3- lymphocyte %leukocyte, CD33 + HLA DR + CD14- Absolute Count, CD33dim HLA DR + CD11b- Absolute Count, CD45 on Monocytic Myeloid-Derived Suppressor Cells and Granulocytic Myeloid-Derived Suppressor Cells Absolute Count were negative correlated with eGFR.

#### MR analysis of cytokine and 731 immune cells on UACR

As shown in supplemental Fig. 2, 1 cytokine IFNg and 22 immune cells feature are associated with UACR. CD123 on CD62L + plasmacytoid Dendritic Cell, CD123 on plasmacytoid Dendritic Cell, CD24 on IgD- CD38- B cell, CD25 + + CD45RA- CD4 not regulatory T cell Absolute Count, CD45RA- CD4 + T cell %, HLA DR + CD4 + T cell %, HVEM on Central Memory CD8 + T cell, Naive-mature B cell Absolute Count, switched memory B cell Absolute Count, CD11c on CD62L + myeloid Dendritic Cell, CD11c on monocyte, CD14- CD16- Absolute Count were positive correlated with UACR. CD27 on switched memory B cell, CD27 on unswitched memory B cell, CD3- lymphocyte %leukocyte, CD33 + HLA DR + CD14- Absolute Count, CD45 on Monocytic Myeloid-Derived Suppressor Cells, CD8 + T cell Absolute Count, Granulocytic Myeloid-Derived Suppressor Cells Absolute Count, CCR2 on CD62L + myeloid Dendritic Cell, CD19 on switched memory B cell, CD19 on unswitched memory B cell were negative correlated with UACR.

### MR sensitivity analysis

In the analysis of the microbiome, 41 cytokines and 731 immune cells were examined in relation to CKD, eGFR and UACR. The MR-Egger intercept and MR-PRESSO P-values were both greater than 0.05, indicating the absence of minimal horizontal pleiotropy and no horizontal pleiotropy was detected (all P_Global.test and P pleiotropy > 0.05) (Table S5 and S6).

### Mediation analyses of potential mediators

After screening for potential intermediate factors, we found 14 microbial taxa and microbial metabolism taxa exerted their effects on CKD through mediation by 10 immune cell phenotypes (Table S4). 10 microbial taxa and microbial metabolism taxa exerted their effects on eGFR through mediation by 10 immune cell phenotypes. Similarly, ten microbial taxa and microbial metabolism taxa exerted their effects on UACR through mediation by nine immune cell phenotypes.

As shown in Table 1, we found that, in MiBioGen dataset, “CD3 on CD28 + CD4-CD8- T cell” and “Naive-mature B cell Absolute Count” exhibited mediation in the causal association between *Coprococcus* and CKD, with a mediation proportion of 7.33 and 6.62(*p* < 0.05). “Naive-mature B cell Absolute Count” exhibited mediation in the causal association between *Lachnospiraceae_*UCG010 and CKD, with a mediation proportion of 6.78(*p* < 0.05). In DMP dataset, “CD28 + CD45RA + CD8 + T cell %”, “CD28 + CD45RA + CD8dim T cell %”, “CD45RA + CD8 + T cell %”, “CD86 on myeloid Dendritic Cell” exhibited mediation in the causal association between *Alistipes indistinctus* and CKD, with a mediation proportion of 1.59, 3.05, 1.50, and 11.72(*p* < 0.05). “CD28 + CD45RA + CD8 + T cell %”, “CD45RA + CD8 + T cell %” exhibited mediation in the causal association between *Alistipes putredinis* and CKD, with a mediation proportion of 1.21 and 1.37 (*P* < 0.05). “CD33 on Granulocytic Myeloid-Derived Suppressor Cells” can both exhibited mediation in the causal association between *Coprobacter*,* Coprobacter fastidiosus* and CKD with a mediation proportion of 14.81 and 14.82 (*p* < 0.05). Moreover, in metabolism, “CD45RA + CD8 + T cell %” can both exhibited mediation in the causal association between superpathway of polyamine biosynthesis I, *starch degradation III* and CKD with a mediation proportion of 1.24 and 0.82 (*p* < 0.05). “HVEM on CD4 + T cell” and “CD45RA on Terminally Differentiated CD8 + T cell” can exhibited mediation in the causal association between sucrose degradation IV sucrose phosphorylase and CKD with a mediation proportion of 7.77 and 15.24 (*p* < 0.05).

**Table 1:** Mediation effect of gut Microbiome on CKD via immune cell.

Exposure database	Exposure	Mediator	Outcome	Direct effect β1	Direct effect β2	Total effect β_total	Mediate effect(95%CI)	*P*-val	Mediation Proportion
MiBioGen	Genus Coprococcus3.id.11,303	CD3 on CD28 + CD4-CD8- T cell	CKD	-0.3545	-0.0282	-0.1364	0.0100 (-0.03,0.023)	0.1290	-7.33%
MiBioGen		Naive-mature B cell Absolute Count	CKD	0.3063	-0.0295	-0.1364	`- 0.0090 (-0.021,0.003)	0.1331	6.62%
MiBioGen	Genus.Lachnospiraceae	Naive-mature B cell Absolute Count	CKD	0.2721	-0.0295	-0.1183	`- 0.0080 (-0.018,0.001)	0.0982	6.78%
MiBioGen	Order.Desulfovibrionales	CD45RA + CD8 + T cell %T cell	CKD	0.2192	0.0044	0.1420	0.0009 (0, 0.002)	0.1423	0.67%
DMP	Species Alistipes_indistinctus	CD28 + CD45RA + CD8 + T cell %	CKD	-0.2395	0.0048	0.0729	´-0.0012 (-0.002,0.000)	0.0033	-1.59%
DMP		CD28 + CD45RA + CD8dim T cell %T cell	CKD	-0.2028	0.0110	0.0729	`-0.0022 (-0.005,0.000)	0.0560	-3.06%
DMP		CD45RA + CD8 + T cell %	CKD	-0.2498	0.0044	0.0729	´-0.0011 (-0.002,0.000)	0.0832	-1.50%
DMP		CD86 on myeloid Dendritic Cell	CKD	-0.1896	-0.0450	0.0729	0.0085 (-0.002,0.019)	0.1032	11.72%
DMP	Species_ Alistipes_putredinis	CD28 + CD45RA + CD8 + T cell %	CKD	-0.3274	0.0048	0.1310	´-0.00159 (-0.003,0.000)	0.0094	-1.21%
DMP		CD45RA + CD8 + T cell %	CKD	-0.4103	0.0044	0.1310	´-0.0018 (-0.004,0.000)	0.0685	-1.37%
DMP	Species_ Lachnospiraceae_bacterium	CD86 on myeloid Dendritic Cell	CKD	-0.1289	-0.0450	0.0404	0.0058 (0.000,0.012)	0.0613	14.36%
DMP	Genus_Coprobacter	CD33 on Granulocytic Myeloid-Derived Suppressor Cells	CKD	-0.5003	0.0243	0.0820	`-0.012 (-0.024,-0.001)	0.0401	-14.81%
DMP	Species_Coprobacter_fastidiosus		CKD	-0.4992	0.0243	0.0818	´-0.0121 (-0.024,-0.001)	0.0401	-14.83%
DMP	Genus_Streptococcus		CKD	0.2985	0.0243	-0.0678	0.0072 (-0.001,0.016)	0.0897	-10.70%
DMP	1.4.dihydroxy.2.naphthoate. biosynthesis.II.plants		CKD	0.2602	0.0243	0.0634	0.0063 (-0.001,0.013)	0.0839	9.97%
DMP		Naive-mature B cell Absolute Count	CKD	0.1390	-0.0295	0.0634	`-0.0041 (-0.009,0.001)	0.1166	-6.47%
DMP	Starch.degradation.III	CD45RA + CD8 + T cell %	CKD	0.0771	0.0044	-0.0408	0.0003 (0.000,0.001)	0.1436	-0.83%
DMP	Sucrose.degradation.IV. .sucrose.phosphorylase	HVEM on CD4 + T cell	CKD	0.4229	-0.0215	0.1167	´-0.009 (-0.020,0.002)	0.1072	-7.78%
DMP		CD45RA on Terminally Differentiated CD8 + T cell	CKD	0.2939	-0.0605	0.1167	´-0.0178 (-0.037,0.001)	0.0687	-15.24%
DMP	Purine.nucleotides.de.novo	IgD on IgD + CD24- B cell	CKD	0.1977	-0.0349	-0.0947	´-0.0070 (-0.016,0.002)	0.1280	7.29%
DMP	Polyamine.biosynthesis.I	CD45RA + CD8 + T cell %	CKD	0.3595	0.0044	-0.1265	0.0016 (0.000,0.003)	0.0747	-1.25%
DMP		CD28 + CD45RA + CD8 + T cell %T cell	CKD	0.2548	0.0048	-0.1265	0.0012 (0.000,0.002)	0.0221	-0.98%

Additionally, the specific results of mediation analysis between microbiome to CKD, eGFR and UACR are presented in Table 1, Table S9 and supplemental Table S10.

## Discussion

As of 2024, there are 3 studies already discussing the causal relationship between the gut microbiome and CKD^21,22,32^. Here, the innovative points of this study have remedied the shortcomings of previous studies: (1) In addition to the MiBioGen consortium database^23^, the latest microbiome dataset named “Dutch Microbiome Project (DMP)”^24^ was also employed for MR analysis in this study. The utilization of both databases can mitigate the risk of reverse causality bias due to the independence of the exposure data. Furthermore, it facilitates the reduction of the impact of common confounders, enhances the statistical power, and improves the generalizability of the findings, thereby strengthening the reliability of the results. (2) The use of DMP database enable us access the causal relationship at species level with MR analysis. (3) The thresholds for selecting casual-effect relationship are based on the adjusted-q-value. Only the q-value were lower than 0.05, we would assume it as the related microbiome feature for association with the CKD, eGFR or UACR. (4) We examined the mediator effect of 41 cytokines and 731 immune cells between the gut microbiome and CKD, as well as its associated functions eGFR and UACR. Given the microbiome’s role as a primary regulator of immunity and inflammation, an analysis of the mediator effect between the gut microbiome and CKD can elucidate whether the microbiome exerts an influence on CKD via the immune system.

It has been established that inflammation represents a significant risk factor for the progression of CKD^33^. The gut microbiome has been identified as a key regulator of immunity and inflammation in a number of diseases, and there is a growing body of evidence indicating that microbiome-immune interactions play an important role in the progression of CKD^34^. Here, we use MR analysis to analysis the causal relationship between microbiome and CKD and its related renal function eGFR and UACR with the mediator effect in 41 cytokine and 731 immune cells. We selected a total of 8 gut microbiota at MiBioGen database and 8 gut microbiotas at DMP database potential have causal association with CKD. We also selected a total of 7 gut microbiotas at MiBioGen database and a total of 7 gut microbiotas at DMP associated with eGFR and 4 gut microbiotas at MiBioGen and 10 microbiotas at DMP were associated with UACR. Moreover, in immune and cytokine base, we selected 3 cytokine and 22 immune cells feature database associated with CKD, IL4 and 18 immune cells features associated with eGFR and IL4 and 18 immune cells features associated with UACR. Furthermore, mediation analysis revealed that 14 microbial taxa and microbial metabolism taxa exerted their effects on CKD through mediation by 10 immune cell phenotypes, derived from the B cell panel, the maturation stages of the T cell panel and the myeloid cells panel. 10 microbial taxa and microbial metabolism taxa exerted their effects on eGFR through mediation by ten immune cell phenotypes (from B cell panel, memory B cell, memory T cell and myeloid cells panel). Similarly, ten microbial taxa and microbial metabolism taxa exerted their effects on UACR through mediation by nine immune cell phenotypes (from B cell panel, T cell panel, memory B cell and myeloid cells panel). We found that some MR analysis results of CKD are on the contrary to the MR analysis results of eGFR. This is in line with our expectation because one of the definitions for CKD is lower eGFR^25^and this underlines our finding.

Recent clinical studies have highlighted significant alterations in the gut microbiota of patients with CKD and IgA nephropathy, for example *Lachnospiraceae*, *Enterobacteriaceae* and certain *Ruminococcaceae*, and decrease in some *Prevotellaceae*, *Bacteroidaceae*, particularly a decrease in beneficial species such as *Lactobacillus johnsonii* and increase level of *Akkermansia muciniphila*^18,35–37^. Targeting *L. johnsonii* has shown potential in modulating the gut–kidney axis and slowing CKD progression via suppressing aryl hydrocarbon receptor (AHR) signal^18^. On the contrary, increased levels of *Akkermansia muciniphila* can facilitate the deglycosylation of IgA, leading to the development of aggravated IgA nephropathy^37^. However, our data did not suggest the relationship between *Lactobacillus johnsonii* and *Akkermansia muciniphila* with CKD. This may be attributed to the cohort analysis was based on generally young participants with a mean age of 58.5 years and a mean follow-up of 5.3 years, which may not be sufficient to detect differences in the risk of incident CKD. Furthermore, the CKD cohort was composed of patients with diabetes, a specific population whose unique characteristics—such as the use of various medications and adherence to specialized diets—could influence the association between the gut microbiome and CKD^25^.

It is notable that this study elucidates the causal relationship between CKD or eGFR and UACR through the analysis of microbiome data in a single microbiome feature across two distinct databases, namely MiBioGen and DMP^23,24^. Interestingly, *Coprococcus*,* Streptococcus* and *Lachnospiraceae* can be found at both databases have causal link with CKD^21,22^. *Desulfovibrionales* and *Desulfovibrionaceae* families are present in the MiBioGen database and have been linked to CKD. Additionally, *Alistipes indistinctus* and *Alistipes putredinis* species have been identified in the DMP and are associated with CKD^21,22,38^. These findings strong indicate the substantial influence of microbiome characteristics on the development of CKD and the advancement of microbiome research. However, an unexpected observation was made: while *Coprococcus*, *Streptococcus*, and *Lachnospiraceae* were identified in both databases, the β values were not in the same direction across two databases. These results can be attributed to the fact that the microbiome GWAS data were not derived from the same population and we found that the GWAS data for MR analysis in two database is different (Table S2). Nevertheless, these findings still indicate that these microbiome features are significantly associated with CKD and renal functions, thereby suggesting a strong causal relationship and reflects the complexity of the relationship between microbes and CKD. Furthermore, our findings indicate that the β value among MR analysis results in CKD with microbiome and immune features is higher than the β value in eGFR and UACR with microbiome and immune features. In CKD cohorts, GWAS of the complementary kidney function marker blood urea nitrogen (BUN) were also used to combined with the eGFR to assess the relevant with kidney function^25^. This suggests that multiple indicators need to be used for diagnostic in CKD.

In the MR analysis of 731 immune cells and 41 cytokines, we found that b-NGF, GROa and SCGFb and 22 immune cells had a causal relationship with CKD, IL4 and 18 immune cells had a causal relationship with eGFR, and IFNg and 22 immune cells were associated with UACR. Previous studies show similar results to our MR analysis in CKD^39^. In our finding, CKD are positive correlated with effect T cell panel (CD28 + CD45RA + CD8 + T cell %, CD39 + CD8 + T cell Absolute Count, CD45RA + CD8 + T cell %T cell, CD28 + CD45RA + CD8dim T cell %T cel) and myeloid panel. This suggests a potential role for these cells in attenuating inflammatory processes within the renal environment. In addition, the presence of CD28 on these cells may signal an aptitude for robust effector functions upon activation^40^. Moreover, we observed that markers from the memory cell panel (BAFF-R on IgD + CD38- unswitched memory B cell, BAFF-R on switched memory B cell, CD27 on switched memory B cell and HVEM on Central Memory CD8 + T cell) are negatively correlated with eGFR and UACR. This suggests that the pathological progression of CKD is a gradual process driven by persistent immune memory dysregulation and explained that CKD is frequently accompanied by a persistent, low-grade inflammatory state.

It is worth noting that order *Desulfovibrionales*, family *Desulfovibrionaceae* at MiBioGen and *Alistipes indistinctus* and *Alistipes putredinis* at DMP both have positive causal link with CKD and class *Deltaproteobacteria* and order *Desulfovibrionales* have negative causal link with eGFR. Previously MR studies have already reported the casual relationship between *Desulfovibrio spp.* and CKD^21,22,38^. However, the studies did not delve into the species level. Here, we suggest that the adverse Impact of *Alistipes indistinctus* and *Alistipes putredinis* to CKD. *Alistipes Spp* has been mentioned the relative abundance is higher in severe acute kidney injury before^41^. There are several potential mechanisms may be involved in between *Alistipes indistinctus*,* Alistipes putredinis* and chronic function. One is that *Alistipes indistinctus* and *Alistipes putredinis* can reduce the sulfites and sulfates obtained from the diet and the sulfated mucopolysaccharides found in mucin, leading to the generation of a cytotoxic compound, hydrogen sulfide^42^. Consequently, hydrogen sulfide serves as a potent inhibitor of the oxidation of SCFAs in cells, thereby establishing a vicious circle of mutually exclusive metabolic interactions between the host and this bacterium^43^. Another is that *Alistipes indistinctus*,* Alistipes putredinis* can potentially contributes to inflammation and epithelium alterations^44^. Our mediator analysis also suggests that CD86 on myeloid Dendritic Cell is the positive mediator effect of *Alistipes indistinctus* to CKD and “CD28 + CD45RA + CD8 + T cell %”, “CD28 + CD45RA + CD8dim T cell %”, “CD45RA + CD8 + T cell %” is the negative effect between *Alistipes indistinctus*,* Alistipes putredinis* to CKD. It suggests that *Alistipes* can activate innate immunity but inhibit adaptive T cell immunity. Nevertheless, further research is required to elucidate the precise role of *Alistipes indistinctus* and *Alistipes putredinis* in renal function and need to be pay attention.

*Coprococcus* have been found that produce the short chain fatty acid (SCFA) and related to human health^45^. There is a still unclear of the role of *Coprococcus* in human health. Some studies declared that the low abundance of *Coprococcus* species has been linked to a higher incidence of inflammatory bowel disease, obesity and diabetes mellitus II^46,47^. Other study has been mentioned that higher *Coprococcus* have been linked with abdominal symptoms^48^. The role of *Coprococcus* in renal function is also uncertain. Here, we found that *Coprococcus* have causal association with CKD and eGFR. However, when comparing the MiBioGen and DMP databases, we observed contrasting results. In the MiBioGen database, there was a negative correlation between *Coprococcus* and CKD, whereas in the DMP database, there was a positive correlation between *Coprococcus* and CKD. There are two possible explanations for this result. One is that it could be due to the different populations in the two databases. The effect of the microbiome on humans can be caused by many factors. It is conceivable that gene-gene interactions and gene-environment interactions may result in analogous alterations in the gut microbiome, yet yield disparate outcomes. The other is that different species of *Coprococcus* have a different role to renal function, even going down to subspecies, it will have a different role to renal function. In addition, we found that *Coprococcus* can exact their role on renal function through “CD3 on CD28 + CD4-CD8- T cell“, “Naive-mature B cell Absolute Count“, “CD33 on Granulocytic Myeloid-Derived Suppressor Cells“. These findings indicate that *Coprococcus* influences kidney function by modulating immune cell activity, particularly in regulating inflammatory responses through T cells, B cells, and MDSCs. As SCFA can be produced by *Coprococcus*, further research regarding to *Coprococcus –* metabolism – immune cell- CKD axis need to be considered.

This was the first study to conduct a large-scale MR analysis of the causal relationships between the gut microbiome, cytokines, immune cell and CKD and it related renal function. There is some limitation in our studies. Firstly, our study only analyzed the European population. Secondly, the cases of chronic kidney disease and renal, were insufficient. Thirdly, we only analyze the mediator role of cytokine and circulating immune cell. the mechanisms how gut microbiota affected the onset of kidney function remained to be studied. Fourthly, we did not account for treatment status or the sex of the patients in the original GWAS data. Although adjusting for such variables directly in MR analyses can lead to collider bias, we acknowledge that these factors may still have an impact on the results, and this should be considered when interpreting our findings.

## Conclusion

Ultimately, the gut microbiome is of significant consequence with regard to the incidence and advancement of CKD and renal function. This study comprehensively assessed the causal relationship between the gut microbiota, inflammatory cytokines, and circulating immune cells in order to identify potential biomarkers that could be used for predicting the prognosis and risk of CKD. The study highlights potential mechanisms and offers new insights into targeted interventions for CKD based on the gut microbiome.

## Electronic supplementary material

Below is the link to the electronic supplementary material.

Supplementary Material 1

Supplementary Material 2

Supplementary Material 3

Supplementary Material 4

Supplementary Material 5

Supplementary Material 6

Supplementary Material 7

Supplementary Material 8

Supplementary Material 9

Supplementary Material 10

Supplementary Material 11

Supplementary Material 12

Supplementary Material 13

## Data Availability

The datasets generated and analysed during the current study are available in MiBioGen consortium database(https://mibiogen.gcc.rug.nl/), GWAS catalog(https://www.ebi.ac.uk/gwas/) and CKDGen Consortium (https://ckdgen.imbi.uni-freiburg.de/).
