# Supplementary figures and images for: Mendelian randomization study revealed a gut microbiota-immune system-kidney junction axis in chronic kidney disease
Source: Sci Rep. 2025 Jul 1;15:21685. doi: 10.1038/s41598-025-05941-x (PMC12216554; doi:10.1038/s41598-025-05941-x)

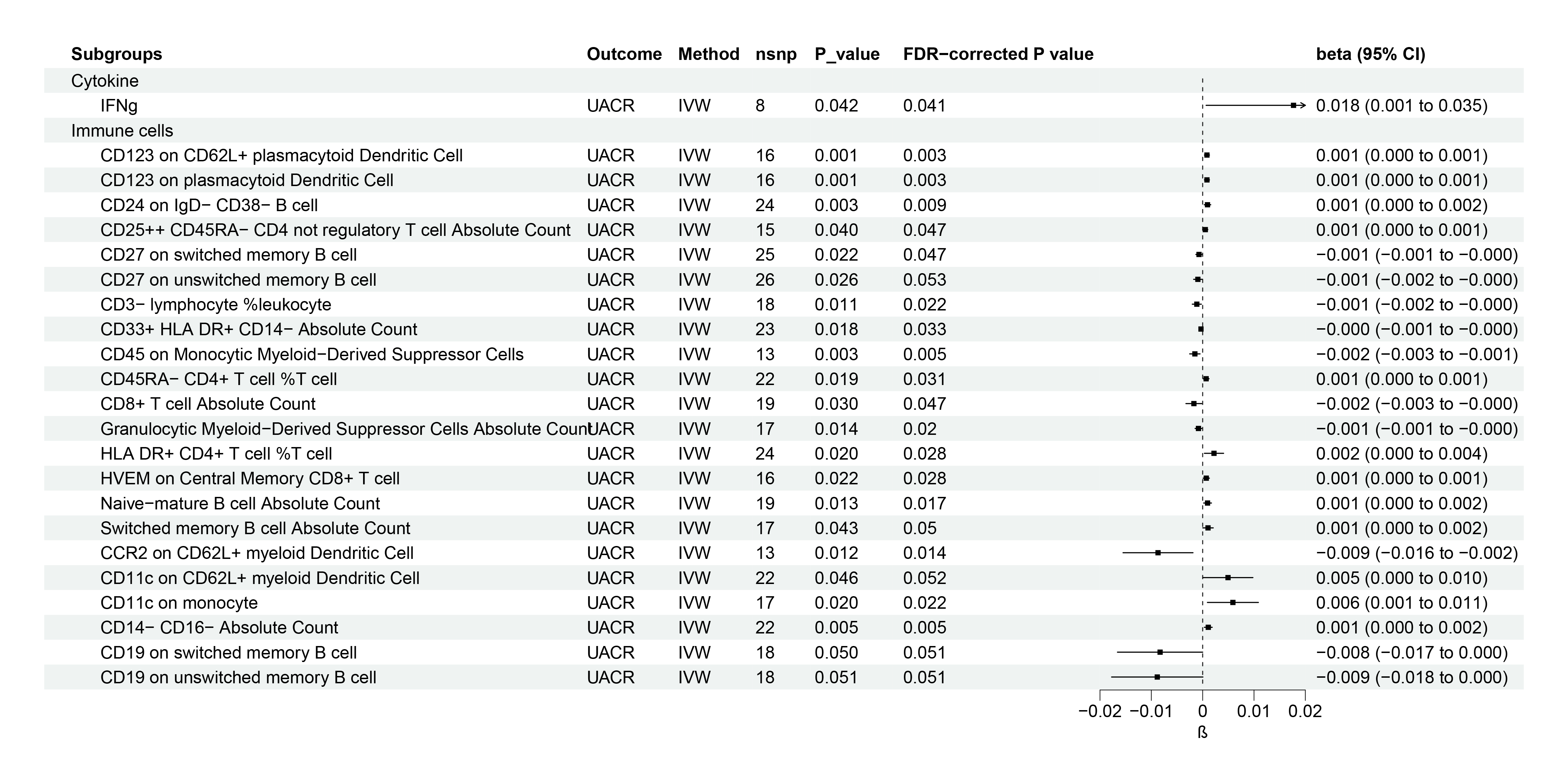

Supplement: Supplementary file 11 — Supplementary Material 11 [file 41598_2025_5941_MOESM11_ESM.tif]

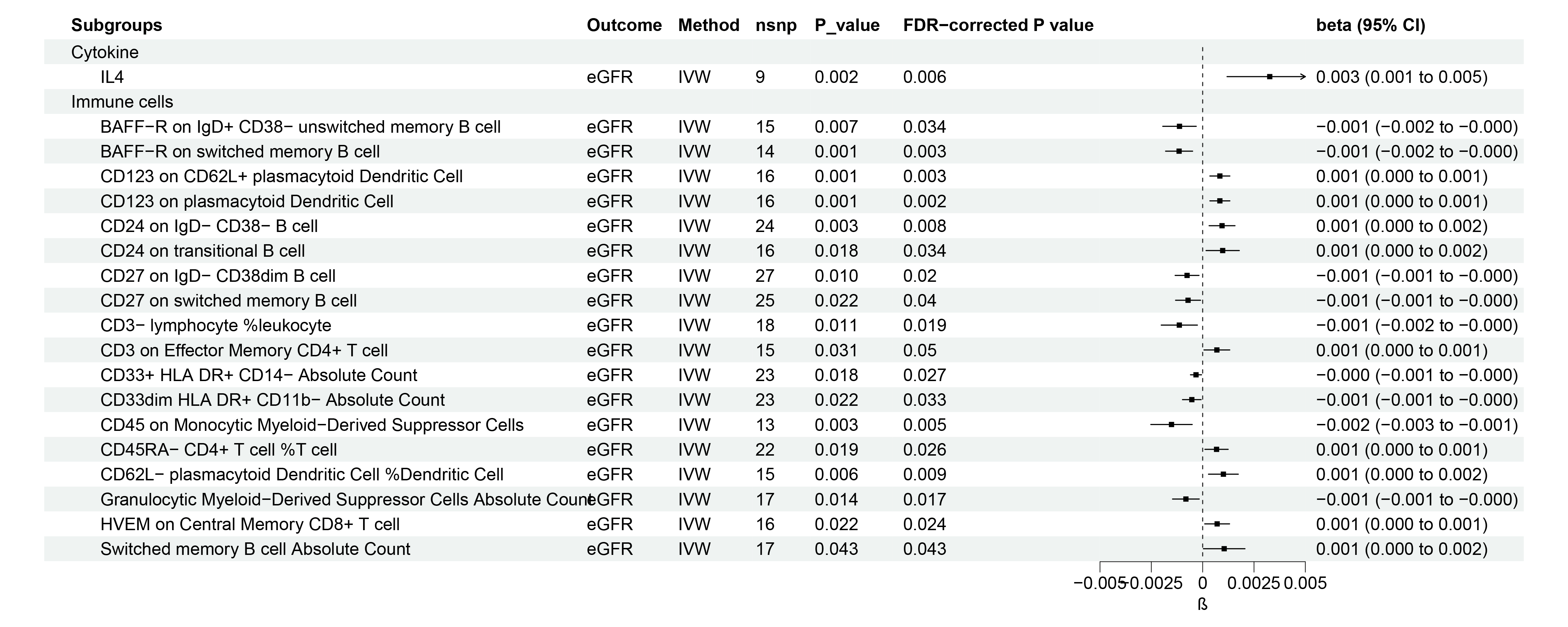

Supplement: Supplementary file 12 — Supplementary Material 12 [file 41598_2025_5941_MOESM12_ESM.tif]
