# Supplementary material for: Mendelian randomization study revealed a gut microbiota-immune system-kidney junction axis in chronic kidney disease
Source: Sci Rep. 2025 Jul 1;15:21685. doi: 10.1038/s41598-025-05941-x (PMC12216554; doi:10.1038/s41598-025-05941-x)
Supplement: Supplementary file 13 — Supplementary Material 13 [file 41598_2025_5941_MOESM13_ESM.docx]

**Supplementary Information**

Supplementary table 1 Description of data on gut microbes, inflammatory cytokines, and chronic disease

Supplementary table 2 Details of the instrumental variables (IV) included in the analysis

Supplementary table 3 Full table of MR analysis results about the casual association between gut microbiome and CKD, eGFR and UACR

Supplementary table 4 Full table of MR analysis results about the casual association between cytokine and immune cell and CKD, eGFR and UACR

Supplementary table 5 Sensitivity analysis results of all MR analysis between gut microbiome and CKD, eGFR and UACR

Supplementary table 6 Sensitivity analysis results of all MR analysis between cytokine and immune cells and CKD, eGFR and UACR

Supplementary table 7 Full table of reverse MR analysis results about the casual association between gut microbiome and CKD, eGFR and UACR

Supplementary table 8 Sensitivity analysis results of reverse MR analysis between gut microbiome and CKD, eGFR and UACR

Supplementary table 9 Mediation effect of gut microbiome and metabolism on eGFR via immune cell.

Supplementary table 10 Mediation effect of gut microbiome and metabolism on UACR via immune cell.

Supplementary figure 1 Results of Bi-directional Univariate Mendelian Randomization on the interplay between the mediators (immune cells and inflammatory cytokines) and eGFR. eGFR: estimated glomerular filtration rate; IVW, inverse variance weighted; CI, confidence interval.

Supplementary figure 2 Results of Bi-directional Univariate Mendelian Randomization on the interplay between the mediators (immune cells and inflammatory cytokines) and UACR. UACR: urinary albumin to creatinine; IVW, inverse variance weighted; CI, confidence interval.
